# Cytoproliferative and Anti-Oxidant Effects Induced by Tannic Acid in Human Embryonic Kidney (Hek-293) Cells

**DOI:** 10.3390/biom9120767

**Published:** 2019-11-22

**Authors:** Pearl O. Perumal, Priscilla Mhlanga, Anou M. Somboro, Daniel G. Amoako, Hezekiel M. Khumalo, Rene M. Khan

**Affiliations:** 1 Discipline of Medical Biochemistry, School of Laboratory Medicine and Medical Science, University of KwaZulu-Natal, Durban 4000, South Africa; 215029604@stu.ukzn.ac.za (P.O.P.); 215063814@stu.ukzn.ac.za (P.M.); kumaloh@ukzn.ac.za (H.M.K.); 2 Biomedical Resource Unit, School of Laboratory Medicine and Medical Sciences, College of Health Sciences, University of KwaZulu-Natal, Durban 4000, South Africa; anou.somboro@gmail.com

**Keywords:** tannic acid, cytoprotective mechanisms, anti-oxidant, pro-oxidant, hek-293 cells, cytotoxic response, enzymes

## Abstract

Tannic acid (TA) portrays a myriad of beneficial properties and has forthwith achieved incessant significance for its cytoprotective qualities in traditional and modern-day medicine. However, TA displays an ambiguous nature demonstrating anti-oxidant and pro-oxidant traits, beckoning further research. Although vast literature on the anti-proliferative effects of TA on cancer cell lines exist, the effects on normal cells remain unchartered. Herein, the cytoproliferative and anti-oxidant effects induced by TA in human embryonic kidney (Hek-293) cells were investigated. Data obtained from the 3-(4,5-dimethyl-2-thiazolyl)-2,5-diphenyl-2H-tetrazolium bromide (MTT) assay demonstrated that TA increased the cell viability and cellular proliferation rate at higher concentrations. Hoechst assay, examining proliferation marker Ki67 supported these findings. DNA fragmentation and oxidative stress-inducers were specifically noted at IC25 and IC50 treatments via biochemical assays. This alluded to TA’s pro-oxidant characteristics. However, the countervailing anti-oxidant defence mechanisms as the endogenous anti-oxidants and phase2 detoxification enzymes were significantly upregulated. Luminometry fortified the anti-oxidant capacity of TA, whereby executioner caspase-3/7 were not activated subservient to the activation of initiator caspases-8 and -9. Thus, proving that TA has anti-apoptotic traits, inter alia. Therefore, TA proved to harbour anti-oxidant, anti-apoptotic, and proliferative effects in Hek-293 cells with its partial cytotoxic responses being outweighed by its cytoprotective mechanisms.

## 1. Introduction

Naturally-derived medicinal plant inhibitors occupied the traditional praxes for centuries and has forthwith achieved incessant significance in modern scientific research. This is due to their minimal toxic side effects when correlated with current adjuvant and drug treatment therapies [1,2]. Among the numerous bioactive plant-gleaned biomolecules, polyphenols remain a focal point considering their ubiquity and beneficial properties [3,4,5]. Polyphenolic molecules have demonstrated improvements in cardiovascular diseases, neurodegenerative conditions, chronic diseases and possesses an influential radical scavenging activity [4,6]. Described as a superfamily possessing numerous health benefits, polyphenols encompass anti-cancer, anti-mutagenic, anti-inflammatory, anti-microbial, anti-tussive, anti-bacterial, anti-septic, and relevant to this study anti-oxidant properties [3,7].

Anti-oxidants are substances imperative in the preclusion of oxidative cellular noxiousness, such as lipid peroxidation and disruption of the deoxyribonucleic acid (DNA) chain. Biological anti-oxidant moieties comprise of enzymes, inhibitors of radical assembly and free-radical quashing agents [8]. Pro-oxidants emanate both endogenous and xenobiotic origins and contribute to the state of oxidative stress by generating reactive oxygen species (ROS) or by precluding the endobiotic anti-oxidant defence systems [9]. Endogenously, pro-oxidants are by-products of aerobic metabolism, serving to oxidise other reactants in a redox reaction [10]. Oxidative stress is generated from a perversion in cellular homeostasis, owing to higher level of oxidants as to anti-oxidants [9]. This ensues a surplus of ROS and reactive nitrogen species (RNS) moreover the detoxifying capacity of local tissues [9,10,11]. Thus, provoking oxidative lesions to membranes, proteins and genes.

Phenolic tannins function to alleviate the unstable and vastly reactive oxygen-free radicals generated during oxidative stress and normal metabolic reactions [8]. Based on their chemical conformation, tannins are categorically segmented into non-hydrolysable tannins (proanthocyanidins) and hydrolysable tannins. The most salient constitution of hydrolysable tannins are ellagitannins and gallotannins (tannic acid) [6]. Tannic acid (TA), abundantly located in oak wood, tea, berries, roots, nuts, galls and wine, is hydrolysed by tannase into its gallic acid (GA) and glucose components [12,13].

The anti-oxidant mechanisms associated with TA suggests an ambiguous nature. For example, it can either serve as a pro-oxidant, inducing DNA damage in the presence of transition metals or as an anti-oxidant, repressing hydroxyl radical generation. Although suggested that the nature of a substance may be pro-oxidant or anti-oxidant depending on its environment, the absolute comprehension of the anti-oxidant nature of TA requires further conclusive research. Due to TA being an astringent and possessing prominent roles in industry, such as dying or tanning leather, it raises the question if TA is chemically toxic to one’s health upon dietary intake. Therefore, the implementation of various methods to evaluate TA in food and environmental analysis is crucial.

Vast studies demonstrated the anti-proliferative responses of TA on cancer cells, however research on normal cell lines remain an unchartered domain. Hence, alluding to the robust anti-oxidant capacity of polyphenols, this study was adopted to investigate the cytoproliferative and anti-oxidant effects induced by TA. Hek-293 cells are a specific, immortalised cell line obtained from human embryonic kidneys which are cultured in vitro. Apart from the kidney’s functionalism as the body’s filter system to toxic components, Hek-293 cells were study-specific due to their reputation of possessing dependable growth and a proclivity for transformation.

## 2. Materials and Methods

### 2.1. Materials

Hek-293 cells were obtained from stores and repropagated for use. Tannic acid was purchased from Sigma Aldrich (Johannesburg, South Africa). All cell culture reagents and low-melting point agarose (LMPA) were procured from Whitehead Scientific (Johannesburg, South Africa). The MTT salt, phosphate buffered saline (PBS), thiobarbituric acid (TBA), malondialdehyde (MDA) and bicinchoninic acid (BCA) were acquired from Capital Laboratory Supplies (Johannesburg, SA). Western blot reagents and anti-Ki67 were purchased from Bio-Rad (Hercules, CA, USA), whilst the remaining antibodies and Promega products were obtained from Anatech (Johannesburg, South Africa). Other reagents utilised were attained from Merck (Darmstadt, Germany) unless stated otherwise. Ethical approval was obtained from the Biomedical Research Ethics Administration (BE469/18).

### 2.2. Cell Culture and TA Treatment

Cryopreserved Hek-293 cells were cultured at 37 °C in a humid atmosphere of 5% CO_2_, using a 25 cm^3^ culture flask. The cells were subsequently submerged in complete culture medium (CCM), which comprised of Dulbecco’s Modified Eagle Medium (DMEM) (10% foetal calf serum, 1% L-Glut and 1% penicillin/ pen-strep-fungizone). Treatments were prepared from 2 mg/mL (1175.64 μM) stocks. Photoprotective precautions were adopted and applied when working with the photosensitive TA.

### 2.3. Cell Viability Assay

The MTT assay was performed to assess the cell viability and cytotoxicity of TA on Hek-293 cells. Cells were seeded in triplicates, at a density of approximately 15,000 cells/well and were cultivated in a 96-well microtiter plate (200 μL). After an overnight incubation (37 °C, 5% CO_2_) to facilitate cell attachment, Hek-293 cells were incubated with a range of TA concentrations (0–1000 μM) for 24 h. Thereafter, the treatment media was discarded, and the cells were re-immersed in 20 μL MTT salt solution (5 mg MTT in 1 mL 0.1M PBS) and 100 μL CCM. After a 4 h incubation (37 °C), 100 μL dimethyl sulphoxide (DMSO) was aliquoted per well and incubated for 1 h (37 °C). The optical density of the formazan product was quantified using spectrophotometry (Bio-Tek μQuant, Winooski, VT, USA) at 570/690 nm. The percentage cell viability was determined from the following Equation (1):

(1) $$\left(\%\;cell\;viability=\frac{average\;OD\;of\;treated\;cells}{average\;OD\;of\;control\;cells}\times 100\right)$$

This was used to generate a concentration-response curve from, which the half maximal inhibitory concentration (IC_50_) was determined. Subsequently, the IC_50_ value was used to generate its associative IC_25_ and IC_75_ values, all of which were utilised for subsequent assays.

### 2.4. Preparation of Cells for Subsequent Assays

Confluent flasks (80%) were treated with TA for 24 h. The cells were washed (0.1 M PBS) and resuspended before being counted and having their volumes adjusted, as per assay requirements. The treatment medium was retained for utilisation in various assays.

### 2.5. Luminometry

#### 2.5.1. Adenosine Triphosphate (ATP) Quantification Assay

Intracellular ATP levels were evaluated using the CellTitre-Glo^®^ kit (Promega, Madison, WI, USA). The treated suspended cells (20,000 cells/well in 50 μL 0.1 M PBS) were pipetted in duplicates into a non-transparent, white 96-well luminometer plate, whilst PBS was used for the blank (50 μL). Thereafter, 50 μL of reagent was added into each well whilst placed on ice. Incubation in the dark for 30 min at room temperature (RT) facilitated the luciferin-luciferase reaction, which produced a luminescent signal that was quantified utilising the Modulus™ microplate luminometer (Turner Bio-systems, Sunnyvale, CA, USA) and expressed as average relative light units (RLU).

#### 2.5.2. Assessment of Caspase Activity

The activities of executioner caspase-3/7 and initiator caspase-8 and -9 were identified using the Caspase-Glo^®^ assay (Promega) to assess the level of apoptosis based on the cleavage of luciferin by luciferase. Initially, 50 μL of treated suspended cells (20,000 cells/well in 50 μL 0.1 M PBS) were pipetted in duplicates into a non-transparent, white 96-well luminometer plate. The blank consisted of 50 μL PBS. The Caspase-Glo^®^-3/7, -8 and -9 reagents were made up as per the manufacturer’s instructions before depositing 50 μL into each well. The plate was then incubated in the dark (30 min, RT). Luminescence, which is proportional to caspase activity, was detected by the Modulus™ microplate luminometer (Turner Bio-systems, Sunnyvale, CA, USA) and expressed in RLU. Thereafter, relative fold-change was calculated to obtain the caspase activity.

### 2.6. Thiobarbituric Acid Reactive Substances (TBARS) Assay

The degree of lipid peroxidation was assessed by the TBARS assay, which quantifies the concentration of malondialdehyde (MDA), a by-product of lipid peroxidation and reflection of oxidative stress. The treatment medium (200 μL per control and treatment) was added into glass test tubes. A negative control (200 μL CCM) and positive control (199 μL + 1 μL MDA) were prepared. Thereafter, 200 μL of 2% phosphoric acid (H_3_PO_4_) and 7% H_3_PO_4_ was added to each tube. However, 400 μL TBA/BHT (butylated hydroxytoluene) was added to each tube except the blank. Instead, 400 μL of 3 mM hydrochloric acid (HCl) was added to the blank (negative control). The pH (1.5) was adjusted by adding 200 μL of 1M HCl upon vortex. Samples were boiled (100 °C, 15 min), then cooled at RT before the addition of 1500 μL butanol. The amalgamation was vortexed before the upper layer was then transferred (200 μL) in triplicates into a 96-well plate. Absorbance was measured using spectrophotometry (Bio-Tek μQuant, Winooski, VT, USA) at 532/600 nm. The average concentration of MDA (μM) was established by dividing the mean replicates by the absorption co-efficient (156 mM^−1^). The absorbance value is directly proportional to the concentration of MDA.

### 2.7. Nitric Oxide Synthase (NOS) Assay

The degree of nitrate and nitrite concentrations in samples, which are indicators of cellular RNS, were assessed using the NOS assay. Approximately, 50 μL of the sodium nitrate standards (0–200 μM) and samples (treatment medium) were pipetted in duplicate into a 96-well microtiter plate. Subsequently, 50 μL vanadium (III) chloride, 25 μL sulphanilamide and 50 μL N-1-Napththyl ethylenediamine dihydrochloride were added in quick succession into each well and thereafter incubated (37 °C, 45 min). Absorbance was measured at 540 nm, with a reference wavelength of 690 nm using spectrophotometry (Bio-Tek μQuant, Winooski, VT, USA) and the RNS concentrations were extrapolated from the standard curve obtained.

### 2.8. Single Cell Gel Electrophoresis (SCGE)/ ‘Comet Assay’

The degree of DNA fragmentation was assessed by the comet assay. Two frosted-end microscopic slides (per control and treatment) were triply layered with 800 μL 2% LMPA; 20,000 cells in 25 μL PBS + 1 μL GelRed™ (Biotium, California, CA, USA) + 300 μL 1% LMPA; 300 μL 1% LMPA, respectively. A coverslip was placed on each individual layer to allow the gel to solidify (4 °C, 10 min) before the addition of a subsequent layer. The final coverslips were removed, and the solidified gels were submerged in cell lysis buffer (2.5M NaCl, 100 mM EDTA, 10mM Tris-Cl (pH 10), 1% Triton X-100 and 10% DMSO) to fragment cell components and disintegrate nucleosomes (4 °C, 1 h). Thereafter, the slides were equilibrated in electrophoresis buffer (1mM Na_2_EDTA (pH 13), 300 mM NaOH) for 20 min to unwind DNA in order to expose any alkali-labile sites appearing as strand breaks. The cells were electrophoresed (25 V, 35 min) and thereafter rinsed with neutralising buffer (0.4 M Tris) 3 times at 5 min intervals. Coverslips were replaced, and cells were viewed using a fluorescent microscope (Olympus IXS1 inverted microscope, Tokyo, Japan) with an excitation wavelength of 510–560 nm and an emission wavelength at 590 nm. Approximately 50 images in total were captured per control/treatment. The average tail lengths were quantified using Soft Imaging System (Life Science-Olympus^©^ Soft Imaging Solutions v5, GmbH, Münster, Germany) by measuring the comet from its head to the tail in µM.

The degree of DNA fragmentation was assessed by the comet assay. Two frosted-end microscopic slides (per control and treatment) were triply layered with 800 μL 2% LMPA; 20,000 cells in 25 μL PBS + 1 μL GelRed™ (Biotium, California, CA, USA) + 300 μL 1% LMPA; 300 μL 1% LMPA, respectively. A coverslip was placed on each individual layer to allow the gel to solidify (4 °C, 10 min) before the addition of a subsequent layer. The final coverslips were removed, and the solidified gels were submerged in cell lysis buffer (2.5M NaCl, 100 mM EDTA, 10mM Tris-Cl (pH 10), 1% Triton X-100 and 10% DMSO) to fragment cell components and disintegrate nucleosomes (4 °C, 1 h). Thereafter, the slides were equilibrated in electrophoresis buffer (1mM Na_2_EDTA (pH 13), 300 mM NaOH) for 20 min to unwind DNA in order to expose any alkali-labile sites appearing as strand breaks. The cells were electrophoresed (25 V, 35 min) and thereafter rinsed with neutralising buffer (0.4 M Tris) 3 times at 5 min intervals. Coverslips were replaced, and cells were viewed using a fluorescent microscope (Olympus IXS1 inverted microscope, Tokyo, Japan) with an excitation wavelength of 510–560 nm and an emission wavelength at 590 nm. Approximately 50 images in total were captured per control/treatment. The average tail lengths were quantified using Soft Imaging System (Life Science-Olympus^©^ Soft Imaging Solutions v5, GmbH, Münster, Germany) by measuring the comet from its head to the tail in µM.

The degree of DNA fragmentation was assessed by the comet assay. Two frosted-end microscopic slides (per control and treatment) were triply layered with 800 μL 2% LMPA; 20,000 cells in 25 μL PBS + 1 μL GelRed™ (Biotium, California, CA, USA) + 300 μL 1% LMPA; 300 μL 1% LMPA, respectively. A coverslip was placed on each individual layer to allow the gel to solidify (4 °C, 10 min) before the addition of a subsequent layer. The final coverslips were removed, and the solidified gels were submerged in cell lysis buffer (2.5M NaCl, 100 mM EDTA, 10mM Tris-Cl (pH 10), 1% Triton X-100 and 10% DMSO) to fragment cell components and disintegrate nucleosomes (4 °C, 1 h). Thereafter, the slides were equilibrated in electrophoresis buffer (1mM Na_2_EDTA (pH 13), 300 mM NaOH) for 20 min to unwind DNA in order to expose any alkali-labile sites appearing as strand breaks. The cells were electrophoresed (25 V, 35 min) and thereafter rinsed with neutralising buffer (0.4 M Tris) 3 times at 5 min intervals. Coverslips were replaced, and cells were viewed using a fluorescent microscope (Olympus IXS1 inverted microscope, Tokyo, Japan) with an excitation wavelength of 510–560 nm and an emission wavelength at 590 nm. Approximately 50 images in total were captured per control/treatment. The average tail lengths were quantified using Soft Imaging System (Life Science-Olympus^©^ Soft Imaging Solutions v5, GmbH, Münster, Germany) by measuring the comet from its head to the tail in µM.

### 2.9. Hoechst Assay

Nuclear arrangement and cell morphology were evaluated in Hek-293 cells treated with TA by marking with Hoechst 33342 (H3570) (Invitrogen™, Eugene, OR, USA). Hek-293 cells in their respectively treated flasks were incubated for 24 h. Subsequent washing with 0.1M PBS occurred three times, followed by fixation (10% paraformaldehyde, 5 min) and then washing again (0.1M PBS). Hoechst solution of 5 μg/mL (Molecular Probes, Eugene, OR, USA) was added before incubation (37 °C, 15 min).

Thereafter, cells were washed (PBS) and five images per replicate were viewed and captured, utilising fluorescent microscopy (Olympus IXS1 inverted microscope, Tokyo, Japan). The excitation wavelength was set at 350 nm with an emission wavelength of 450 nm. Magnification was carried out at 200 ×. Cellular morphology and nuclear arrangement were analysed using Soft Imaging System (Life Science-^©^Olympus Soft Imaging Solutions v5).

### 2.10. Western Blotting

Western blotting was utilised to evaluate the protein expression of superoxide dismutase (SOD2), nuclear factor (erythoid-derived 2)-like 2 (Nrf2), glutathione peroxidase (Gpx1), heat shock protein (HSP70) and Ki67. Crude protein of control and treated cells were extracted on ice (15 min) using 200 μL Cytobuster™ reagent (Novagen, San Diego, CA, USA) which was supplemented with protease and phosphatase inhibitors (Roche, Mannheim, Germany). Cell lysates were centrifuged to obtain crude proteins, which were quantified using the BCA assay (Sigma-Aldrich, Darmstadt, Germany) and thereafter standardised to 0.9 mg/mL.

Samples were boiled in Laemmli buffer (dH_2_O, 0.5M Tris-HCl (pH 6.8), 3% glycerol, 10% SDS, 12% β-mercaptoethanol, 1% bromophenol blue), loaded (25 μL) into sodium dodecyl sulphate-polyacrylamide gel electrophoresis (4% stacking; 10% resolving) and electrophoresed with the Bio-Rad compact power supply (150 V, 1.5 h). Separated proteins were electro-transferred onto nitrocellulose membranes using Blot^®^ Turbo Transfer system (Bio-Rad) (2.5 mA, 30 min) and then blocked with 5% bovine serum albumin (BSA) in Tris buffer saline (TTBS) (0.5% Tween20, dH_2_O, KCl, NaCl, Tris, pH 7.5) for 2 h.

Membranes were subsequently immune-probed and incubated with primary antibody [SOD2 (13141), Nrf2 (12721), Gpx1 (3286) and HSP70 (46477), Ki67 (HCA053); 1:1000 dilution in 5% BSA; 1 h. After overnight incubation (4 °C), membranes were washed with TTBS (10 min, 5 times) and probed with horseradish peroxidase (HRP) conjugated secondary antibodies (SOD2, Nrf2 and Gpx1 [anti-rabbit IgG, 7074S], HSP70 and Ki67 [anti-mouse IgG, 7076]; 1:2500; 2 h). Membranes were washed (TTBS) and protein bands were viewed using the Clarity Western ECL substrate (Bio-Rad) detection reagents. Images were captured utilising the Molecular Imager^®^ Chemidoc™ XRS+ Bio-Rad imaging system. Membranes were quenched using 5% hydrogen peroxide (H_2_O_2_), blocked in BSA and rinsed (TTBS) before being probed for β-actin (AbD12141) to normalise the protein expression. The relative band density (RBD) was quantified using Image Lab™ 6.0.1 Software (Bio-Rad, California, CA, USA) and normalised against the housekeeping protein for each sample.

### 2.11. Statistical Analysis

Statistical analysis was performed using GraphPad Prism v5.0 software (GraphPad Software Inc., La Jolla, CA, USA). Statistical significance was assessed using the unpaired t-test with Welch’s correction (data expressed as mean ± standard deviation (SD)) or the one-way analysis of variance (ANOVA) in association with the Bonferroni test for multiple group comparison. The data obtained was deemed statistically significant with a 95% confidence interval and *p* value < 0.05.

## 3. Results

### 3.1. Mitochondrial Productivity

To evaluate the effect of TA on the mitochondrial yield of Hek-293 cells; the cell viability and intracellular ATP levels were measured.

#### 3.1.1. Cell Viability Assay

**Figure 1:**
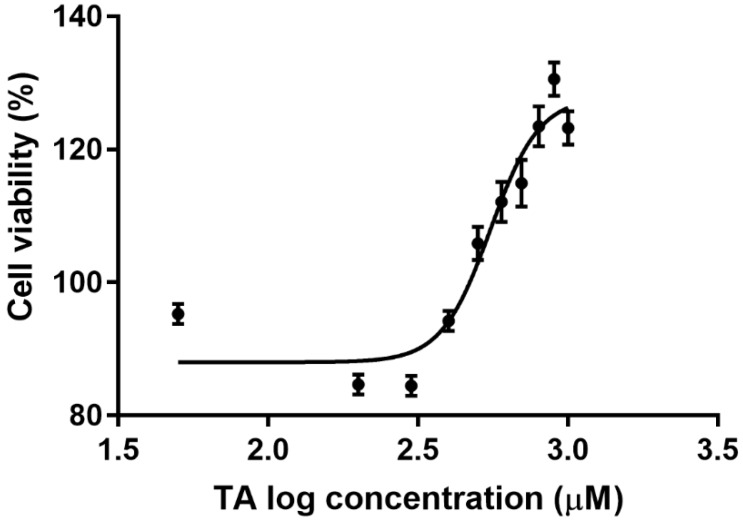
The effect of tannic acid (TA) on Hek-293 cell viability. TA induced a characteristic increase in the viability of Hek-293 cells following a 24 h treatment. A linear regression analysis determined the IC_50_ of TA to be 8.9 µM and the data obtained is represented as a percentage of viable cells relative to the untreated control. Higher concentrations displayed a higher rate of cell proliferation. TA: tannic acid.

The MTT assay was used for the quantification of TA cytotoxicity in Hek-293 cells (Figure 1). A dose-response curve was generated from serially diluted TA concentrations (0–1000 µM) over a 24 h period. A linear regression analysis allowed for the determination of an IC_50_ value (8.9 µM), from which the IC_25_ and IC_75_ values of 4.4 µM and 13.3 µM were generated respectively. These concentrations were utilised as treatments in succeeding assays. Initial concentrations exhibited a slight decrease in cell viability, however not below 85%. After 300 µM, the cell viability began to increase in a dose-dependent manner, with the highest viability obtained being 128%. Therefore, higher concentrations amplified cell proliferation in Hek-293 cells.

#### 3.1.2. Intracellular ATP Levels

**Figure 2:**
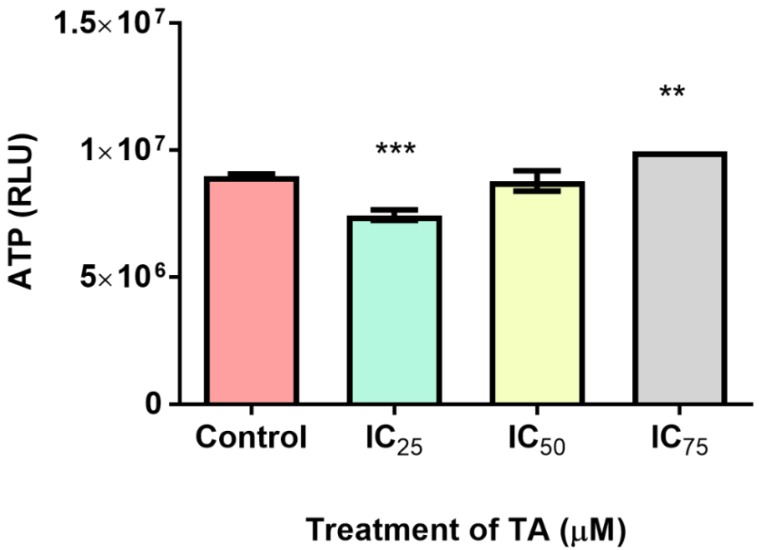
Levels of adenosine triphosphate (ATP) in the untreated control vs. treated Hek-293 cells. Tannic acid decreased ATP levels at IC_25_ (1.2-fold) and increased ATP levels at IC_75_ (1.1-fold) relative to the control (*** *p* < 0.0001, ** *p* < 0.05).

Intracellular ATP levels were quantified via luminometry (Figure 2). TA-induced ATP levels displayed a significant 1.2-fold decrease at IC_25_ (7,448,000 ± 119,800 RLU; *** *p* < 0.0001) and a significant 1.1-fold increase at IC_75_ (9,955,000 ± 2887 RLU; ** *p* < 0.05). Treatment at IC_50_ (8,795,000 ± 233,100 RLU; *p* = 0.5095) did not exhibit any significant change in relation to the control (8,984,000 ± 47,570 RLU).

### 3.2. Oxidative Stress

**Figure 3:**
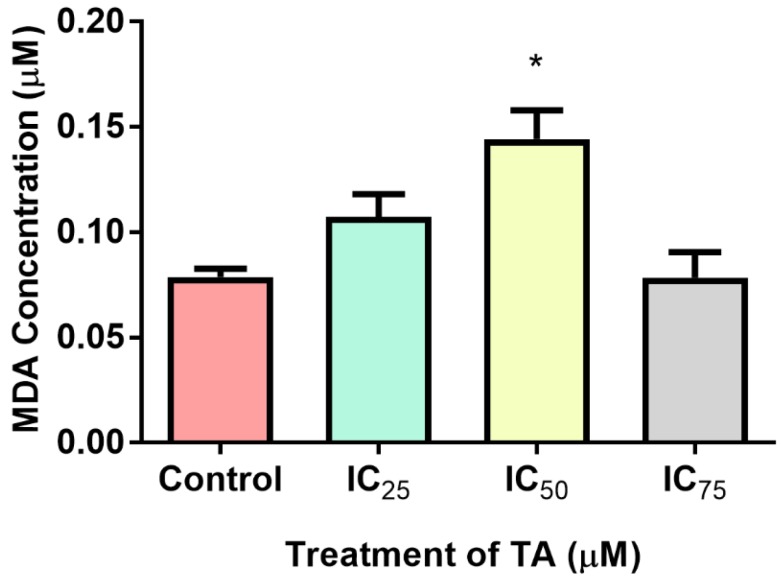
Malondialdehyde (MDA) concentration of Hek-293 cells at IC_25_, IC_50_ and IC_75_ treatments. Tannic acid induced oxidative stress at IC_25_ (1.3-fold), with a 1.8-fold rise at IC_50_, as indicated by the elevated MDA concentrations. ROS production remained almost unchanged at IC_75_ relative to the control. (* *p* < 0.05).

Lipid peroxidation via ROS was used as an indicator of oxidative stress by evaluating the levels of extracellular MDA (Figure 3). MDA levels remained almost equivalent to the control at IC_75_ (0.07837 ± 0.007014 µM; *p* = 0.9681) but exhibited a 1.3-fold increase at IC_25_ (0.1072 ± 0.006301 µM; *p* = 0.0512). However, MDA concentration increased significantly by 1.8-fold at IC_50_ (0.1442 ± 0.007869 µM; *p* = 0.0153) as compared to the control (0.0787 ± 0.002318 µM).

### 3.3. Nitrosative Stress

**Figure 4:**
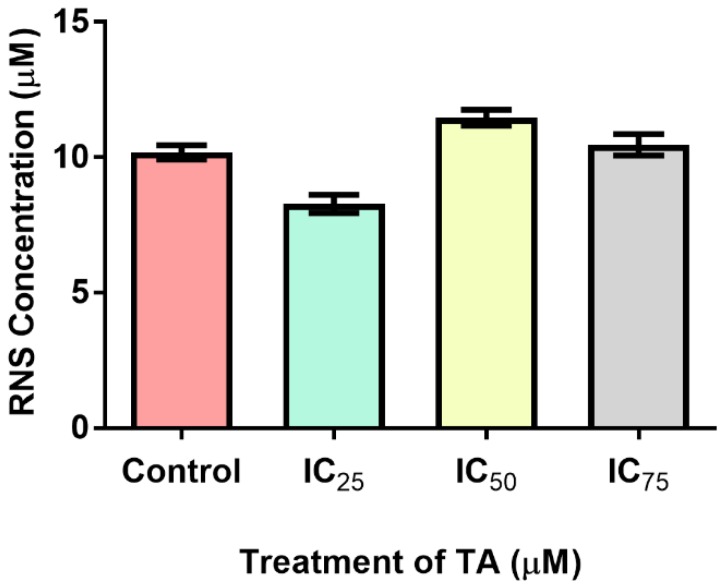
Nitrosative stress induced in Hek-293 cells based on varying TA treatments. Generation of RNS was non-significantly increased at IC_50_ (12.5%) and IC_75_ (2.6%), whilst a non-significant decrease occurred at IC_25_ (18.7%) relative to the control. RNS: reactive nitrogen species.

Nitrosative stress was assessed by quantifying the extent of reactive nitrogen species (RNS) generated (Figure 4). Levels of RNS displayed non-significant changes at the various treatments when compared to the control (10.19 ± 0.1850 µM). The IC_25_ decreased by 18.7% (8.280 ± 0.2400 µM; *p* = 0.1004), IC_50_ increased by 12.5% (11.46 ± 0.2100 µM; *p* = 0.1376) and IC_75_ increased minimally by 2.6% (10.46 ± 0.2800 µM, *p* = 0.5630).

### 3.4. Anti-Oxidant Response and Phase 2 Detoxification

**Figure 5:**
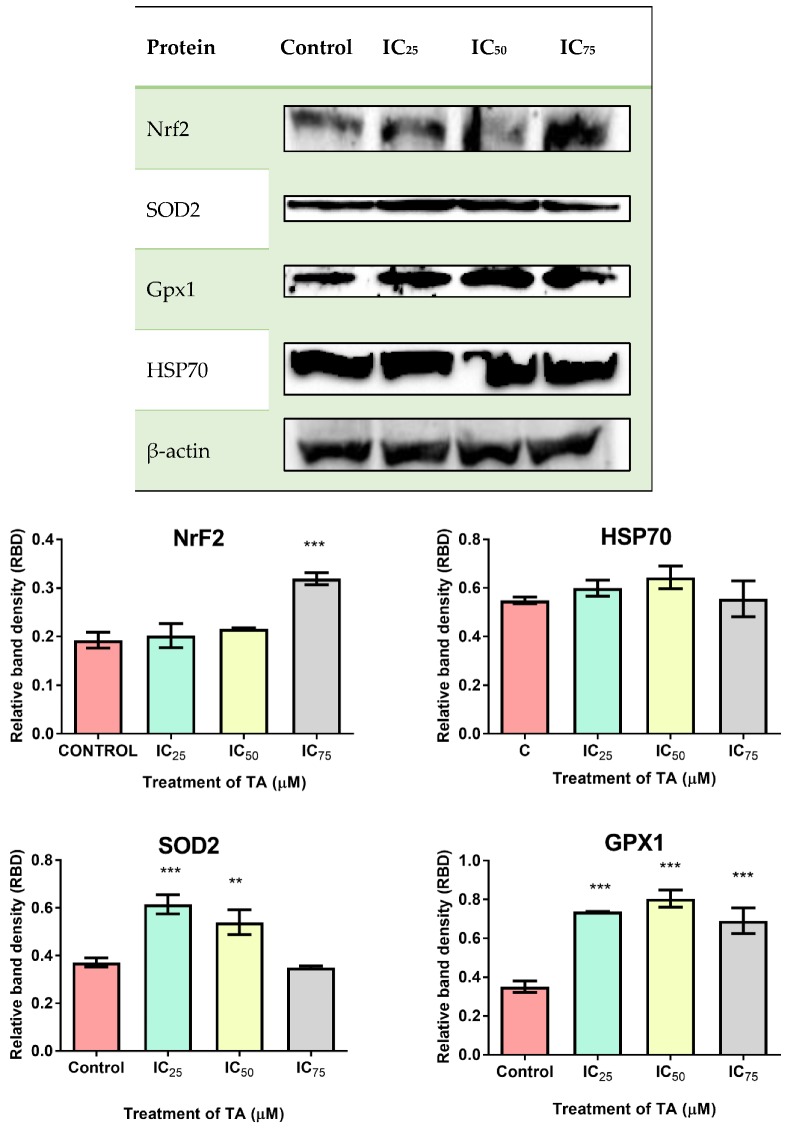
Differential expression of anti-oxidant and phase 2 detoxification response proteins (Nrf2, SOD2, Gpx1 and HSP70) in Hek-293 cells following treatment at IC_25_, IC_50_ and IC_75_ for 24 h. All treatments exhibited an upregulation in protein expressions (*** *p* < 0.0001, ** *p* < 0.05).

Western blotting was performed to assess the effect of TA on the relative protein expression of cellular anti-oxidant systems (SOD2, Nrf2, Gpx, HSP70) (Figure 5). When compared to the control, SOD2 displayed an upregulation of 1.7-fold at IC_25_ and 1.5-fold at IC_50_ treatments, with IC_75_ being non-significantly downregulated. A significant upregulation was observed for Gpx1 (IC_25_: 2.1-fold, IC_50_: 2.3-fold, IC_75_: 2.0-fold), whilst HSP70 was non-significantly upregulated (IC_25_: 1.1-fold, IC_50_: 1.2-fold, IC_75_: 1.0-fold) at all treatments. Nrf2 exhibited an elevation in expression in all treatments, with a significant 1.7-fold upregulation at IC_75_.

### 3.5. Caspase Activation

**Figure 6:**
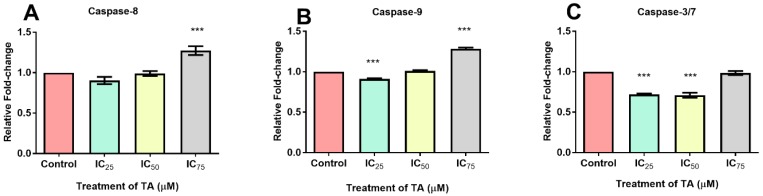
The effect of TA on initiator caspases (**A**,**B**) and executioner caspases (**C**) in Hek-293 cells. Both (**A**) caspase-8 and (**B**). caspase-9 were downregulated at IC_25_ and IC_50_ treatments, whilst IC_75_ treatments significantly increased initiator caspase activation relative to the control. (**C**) Caspase 3/7 were downregulated at all treatments as compared to the control (*** *p* < 0.0001). Data is expressed as relative fold-change.

Luminometry detected the activity of executioner caspase-3/7 and initiator caspases -8 and -9 in the presence of TA. The data in Table 1 and Figure 6 illustrates that at IC_25_ caspase activity had decreased, with caspase-8 and -9 being significantly reduced. The IC_50_ treatment exhibited a decrease by 29.4% at caspase-3/7, with a minimal elevation by 1% and 0.4% at caspase-8 and -9, respectively. Cells treated at IC_75_ showed a 1.7% decrease for caspase-3/7 and significant elevations for capase-8 (27.1%) and -9 (28.5%).

### 3.6. DNA Fragmentation

**Figure 7:**
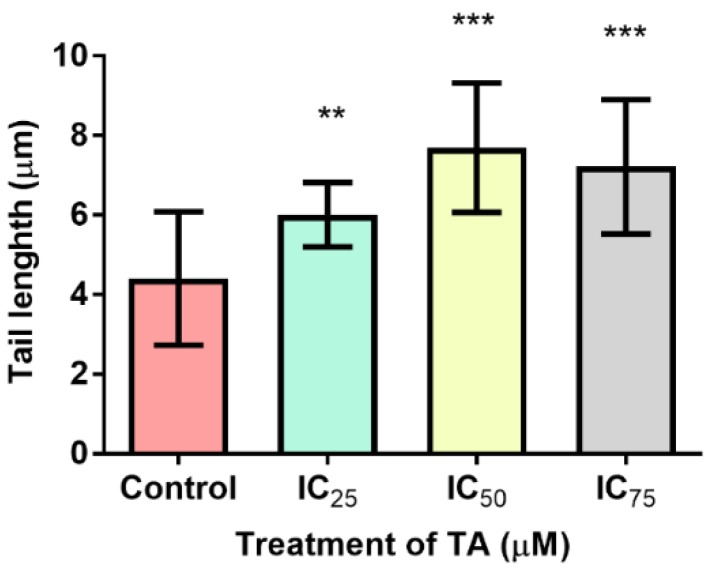

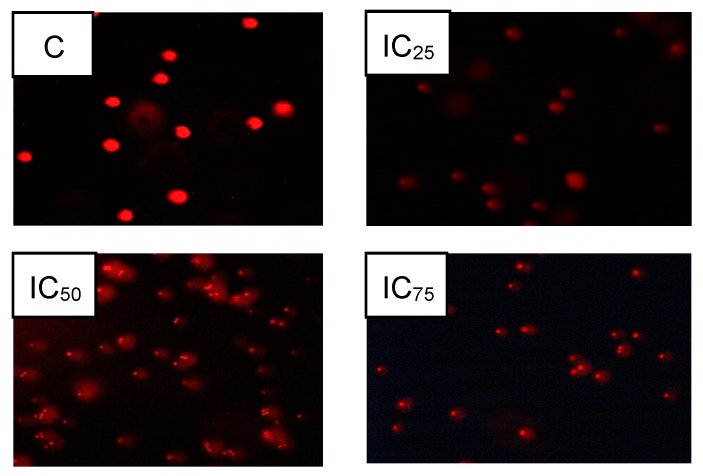
Degree of DNA fragmentation induced by TA in Hek-293 cells. DNA damage was increased at both IC_50_ and IC_75_ (63.8%) treatments relative to the control (*** *p* < 0.0001), with a vast 74.7% elevation in DNA fragmentation noticed at IC_50_ in Hek-293 cells. A 36.4% increase in DNA damage was exhibited at IC_25_ treatment (** *p* < 0.05) relative to the control. TA: tannic acid, C: control.

The SCGE assay was performed to deter mine the degree of DNA fragmentation in TA treated cells (Figure 7). There was a significant increase in comet tail lengths at all treatments [IC_25_: (6.013 ± 0.1146 µM); (IC_50_: 7.697 ± 0.2297 µM); (IC_75_: 7.217 ± 0.2389 µM)] as compared to the control (4.407 ± 0.2377 µM).

### 3.7. Analysis of Proliferation

**Figure 8:**
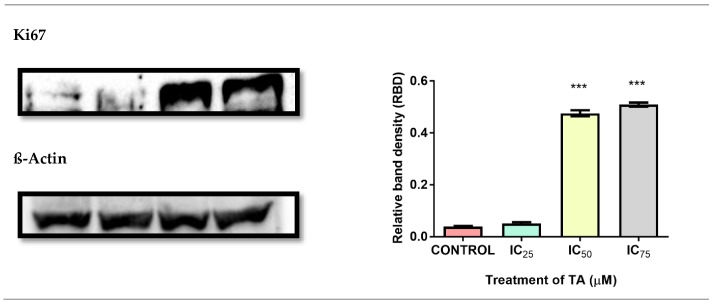
Differential expression of proliferation marker Ki67 in Hek-293 cells following treatment for 24h. All treatments exhibited an upregulation in protein expression, inferring an increased rate of proliferation. (*** *p* < 0.0001).

Western blot results inferred that Ki67 (marker of proliferation) demonstrated a concentration-dependent increase in cell growth at all treatments, with a significant upregulation at IC_50_ and IC_75_ treatments (12.3-fold and 13.2-fold respectively) (Figure 8).

### 3.8. Hoechst Assay

**Figure 9:**
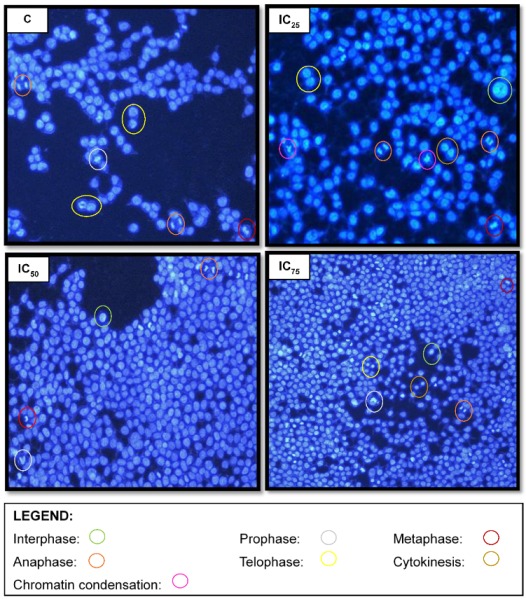
Nuclear arrangement and morphology of untreated Hek-293 cells vs. tannic acid treated Hek-293 cells (200×). Tannic acid induces proliferation in a concentration-dependent manner. C: control.

Hoechst staining assay detected the nuclear arrangement, morphology and various stages of mitosis in Hek-293 cells. There were minor features of the late stages of apoptosis noticed (chromatin condensation). However, expansion in the cell population and a higher rate of mitosis was observed at increased concentrations. This assay illustrated an escalation in cell density and viable cells that underwent various stages in mitosis in both the untreated control and at different treatment concentrations (Figure 9).

## 4. Discussion

Plant-derived polyphenolic phytochemicals possess a ubiquitous presence in our nutritional consumption and exhibit a broad spectrum of beneficial properties [4,11]. Among the plethora of polyphenols that exist, tannic acid’s utilisation in traditional medicine dates back for generations, from treating poisonous substances to the amplification of the therapeutic potency of herbal medicine [1]. Prevailing research has noted its anti-carcinogenic, anti-microbial, anti-mutagenic, anti-inflammatory, anti-microbial, anti-allergic and anti-oxidant efficacy [3,7,14,15]. However, the anti-oxidant mechanisms associated with TA demonstrates an ambiguous nature as it can either serve as a pro-oxidant inducing DNA damage (Figure 7), or as an anti-oxidant repressing hydroxyl radical generation by chelating transition metals [1,6].

In the present study, TA demonstrated a robust proliferative effect on Hek-293 cells with an IC_50_ of 8.9 µM (Figure 1). The MTT assay evaluates cell viability based on the quantity of reducing equivalents generated by metabolically active cells [16,17]. The upward-sloping sigmoidal curve that was generated in correlation with the upregulation of proliferation marker-Ki67 (Figure 8), in a concentration-dependent manner, justified the proliferative effect of TA. Ki67 is exclusively associated with cellular proliferation, being detected solely in the nucleus during interphase and repositioning to the surface of chromosomes during mitosis [18]. Thus, it is only present in the active stages of the cell cycle, except the quiescent stage (G_0_). The morphological changes illustrated by the Hoechst analysis images validates this phenomenon (Figure 9).

Since insufficient research on the cytoproliferative effects of TA on immortalised cell lines exist, the exact mechanism favouring cell growth is unknown. However, it is important to consider the biological nature of the Hek-293 cell line itself. It is characterised as telomerase positive, containing the Simian vacuolating virus 40 T- antigen (SV40 Tag), which perturbs retinoblastoma and p53 proteins [19,20]. This results in the cell’s exit from the G_1_ phase of the cell cycle into the S phase, thus promoting DNA replication and mitosis (Figure 9) [20]. However, since transformed cells possess a copious supply of glycolytic enzymes, coupled with DMEM providing glucose and glutamine as its prime carbon and energy source-metabolic activity would increase as such [21]. The aforementioned presupposes the enhancement of TA on these mechanisms.

TA is a deca-galloyl glucose molecule which hydrolyses into its gallic acid (GA) and glucose components [8]. GA is further metabolised to pyrogallol, which may contribute to energy metabolism by its degradative end-product, pyruvate [22,23]. L-glutamine obtained from CCM, is utilised to generate the pivotal tricarboxylic acid cycle (TCA) intermediate, α-ketoglutarate [24]. In addition to the pyruvate generated, α-ketoglutarate would serve to propel the progression of the TCA cycle, eventuating a high energy expenditure by increasing ATP.

Mitochondria are eminent for their functionalism in ATP production via the contribution of the electron transport chain and ATP synthase during oxidative phosphorylation (OXPHOS). However, it also favours implications in signalling, proliferation, differentiation and the regulation of the cell cycle and metabolism [25]. Ergo, an accretion in cellular proliferation aggrandises mitochondrial quantity and yield, suggested that elevated rates of glycolytic ATP generation, may magnify cytosolic ATP/ADP ratio but may dampen mitochondrial ATP output as a result from the foreshortened ADP levels [21]. This allusion to the Warburg effect has had prevailing modicums of validity, a priori, as demonstrated that proliferating cells possess increased glycolytic rates for the pathways executed in the mitochondria [26]. This cogitates that a higher ATP yield would be required to compensate for a status of proliferative desideratum, as illustrated at IC_50_ and IC_75_ treatments of this study’s findings (Figure 2). However, the intracellular ATP levels decreased at IC_25_. This may be attributed to the insignificant increase in proliferation (Figure 8) but is justified by Birkeet et al. in 2011 in that signalling and energy-dependent mechanisms may be reduced upon introduction into a transitional state and may not accompany mitochondrial expansion [27]. However, IC_25_ did elevate the levels of ROS (Figure 3). This could be justified by, in which he acknowledged that polyphenolic pro-oxidant effects are beneficial to a certain extent, as the introduction of moderate oxidative stress, increases the anti-oxidant defences (Figure 5) and ‘xenobiotic-metabolism enzymes’, rendering cytoprotection [28].

Conventional metabolic conditions engender the rhythmic production of ROS and other free radicals for the contribution toward physiological functions such as ATP generation, catabolism, anabolism and the co-existing redox cycles [9,10]. Excessive omnipresence of ROS through biological endogenous or exogenous factors results in the imbalance of oxidants in relation to anti-oxidants, begetting oxidative stress [10]. ROS includes the mitochondrial generated superoxide anion (O_2_^·^), hydroxyl free radical (OH^·^) and hydrogen peroxide (H_2_O_2_) [9]. The O_2_^·^ radical reacts with nitric oxide producing peroxynitrites, which is fomented by reactive aldehydes such as MDA [10]. TA elevated the concentration of MDA, a lipid peroxidation by-product and marker of oxidative stress, at IC_25_ and IC_50_ (Figure 3). RNS concentrations were scantily elevated at higher treatments and demonstrated a decrease at IC_25_ (Figure 4). The elevated levels of ROS can be attributed to the increase in OXPHOS activity due to a spike in cellular metabolism. The quantity and form of ROS and RNS species, exposure period, anti-oxidant capacity and metabolites render various responses- including proliferation and apoptosis [9].

Anti-apoptotic effects induced by TA were observed for all treatments. The IC_25_ demonstrated a significant decrease in the measure of initiator caspases-8 and-9 (Figure 6A,B). Furthermore, whilst the IC_75_ was significantly elevated, there was no subsequent activation of the executioner caspases-3/7 noted (Figure 6C). The dependency of caspase-9 activity on cytochrome c (an iron metalloprotein) may offer an explanation to the trend seen at IC_25_. The forfeiture of iron from cytochrome c during TA chelation may hamper the establishment of an apoptosome, thus reducing caspase-9 activity. Apaf-1 is dependent on ATP for cytochrome c binding but due to the depletion of ATP at IC_25_ (Figure 2), caspase-9 activity is hindered.

The inhibitors of apoptosis proteins (IAP) precludes caspases, preventing apoptosis from being executed. Constitutionally of IAPs, is the 1-3 BIR domain which may fold into zinc-binding formations [29,30]. X-linked IAP (XIAP) is an omnipotent member of the IAP family, whose BIR-2 domain binds to the amino-terminal vestige of caspase-7 provoking its inhibition, whilst the linker portion is accountable for the exclusive inhibition of caspase-3 (Figure 6C). The fundamental mechanism which represents XIAP’s anti-apoptotic potential is the activity of E3 ligase of the really interesting new gene (RING) finger domain, which ensues the ubiquitination of caspases-3/7 [30]. Although there were no signs of apoptosis occurring, the comet assay did reveal that DNA fragmentation was present (Figure 7). This is consistent with the increase in free radicals (Figure 3 and Figure 4), resulting in TA causing single and double stranded DNA lesions as exhibited in a study conducted by Gray et al. [31] Furthermore, caspase-independent systems could be a subservient cause for the above. Heat shock protein 70 is a powerful ATP-dependent chaperone possessing cytoprotective properties and is responsible for cell signaling and the prevention of cellular death [32]. Heightened levels of HSP70 (Figure 5), permits the cell to prevail in an environment of oxidative stress. One of its cytoprotective traits is the disbarring of apoptosis. It is a precluding regulator for the intrinsic apoptotic pathway, in which it may block apoptosis at the pre-mitochondrial level (inhibiting stress signals), at the mitochondrial stage (by inhibiting Bax translocation) and at the post-mitochondrial level (interacting with Apaf-1 or caspase-3 cleavage proteins) [32]. In addition to the aforementioned traits, it facilitates the translocation of precursor proteins into the mitochondria whilst modulating regulatory proteins [32] HP70 levels are directly proportional to the regulation of Nrf2 [33]. Phase 2 detoxification enzymes are imperative in the defence system toward oxidative stress and require the detachment of Nrf2 from Kelch-like ECH associating protein-1 (Keap-1), in order to translocate to the nucleus and transcribe anti-oxidant response genes [16]

This study demonstrates that there was an upregulation of Nrf2 at all treatments (Figure 5), which implies that the anti-oxidant response system of Hek-293 cells was activated [34]. Suggests that Nrf2 is vital for mitochondrial integrity, especially in times of oxidative stress. Subservient to the Nrf2 upregulation was an upregulation of SOD2 at all treatments (Figure 5). Nrf2 transcribes for the SOD2 gene, a mitochondrial detoxification molecule, which catalyses the conversion of O_2_^·^ to H_2_O_2_ [16]. This regulates cellular homeostasis and promotes the protective anti-oxidant defence systems within cells. As a result of the catalytic conversion by SOD2, the H_2_O_2_ levels would have elevated, thus activating the expression of Gpx1. Cytoplasmic selenoprotein, Gpx1, reduces H_2_O_2_ to form water [16]. Figure 5 illustrates the significant increase in Gpx1 expression for all treatments, contributing to the anti-oxidant defence mechanisms. Therefore, this study has demonstrated that the fortification of renal anti-oxidant defence systems by an exogenous anti-oxidant (TA), serves as a cytoprotective approach in protecting the kidney from oxidative lesions. These results correlate with the findings of Akomolafe et al. [35].

## 5. Conclusions

Literature suggests that TA is a potent anti-oxidant, inter alia, which is validated by the cascading activation of anti-oxidant defence systems in the present study. However, TA has displayed both cytoprotective and partial cytotoxic characteristics by exhibiting both anti-oxidant and pro-oxidant features. Although TA induced ROS formation and DNA fragmentation, its anti-oxidant capacity is more than able to counteract and detoxify these free radicals and their accompanying fatalities. Thus, maintaining cellular integrity, negating nephrotoxicity and stimulating proliferation in Hek-293 cells. Future studies should consider a time-dependent approach to exclusively evaluate the ambiguous oxidant nature of TA. More so, whilst most research on polyphenols target in vitro cancer cell lines, it is imperative to evaluate the effects on normal cell lines and in vivo models. The dietary TA consumption in humans remain a grey area in research which needs to be considered. This is imperative for therapeutic usage, as one must be aware of their safe and toxic levels. If this is accomplished, then the beneficial traits that TA harbours will be at the forefront of alleviating certain conditions and diseases. Therefore, TA has the potential to serve as an indispensable contributor to the pharmaceutical, nutraceutical, medicinal and cosmetic fields.

## Figures and Tables

**Table 1:** Caspase activity in tannic acid (TA) treated Hek-293 cells.

Caspase	Mean ± SD (RLU)			
	Control	IC_25_	IC_50_	IC_75_
-8	(1,002,000 ± 32,790)	(903,500 ± 3408) *p* =< 0.05	(991,900 ± 16,860) *p* = 0.8088	(1,274,000 ± 12,220) *p* =< 0.0001
-9	(2,712,000 ± 11,310)	(2,463,000 ± 5447) *p* =< 0.0001	(2,723,000 ± 9690) *p* = 0.5141	(3,486,000 ± 9710) *p* =< 0.0001
-3/7	(130,000 ± 5356)	(93,000 ± 3083) *p* =< 0.0001	(91,810 ± 1493) *p* = 0.1820	(127,800 ± 3484) *p* = 0.7520

Data represented as mean ± the respective standard deviation in relative light units.
